# Localisation endobronchique d'une leucémie aiguë lymphoblastique de phénotype T

**DOI:** 10.11604/pamj.2015.21.5.6725

**Published:** 2015-05-04

**Authors:** Hafsa Sajiai, Siham Fikal, Hind Serhane, Salma Aitbatahar, Lamyae Amro, Abdelhaq Alaoui Yazidi

**Affiliations:** 1 Service de Pneumologie, Hôpital Arrazi, CHU Mohammed VI, FMPM, Labo PCIM, UCA, Marrakech, Maroc

**Keywords:** Leucémie aigue lymphoblastique, endobronchique, bronchoscopie, Acute lymphoblastic leukemia, endobronchial, bronchoscopy

## Abstract

La localisation endobronchique des leucémies aigues lymphoblastiques est exceptionnelle, de rares cas ont été rapportés dans la littérature. Nous rapportons le cas d'une localisation endobronchique d'une leucémie aigue lymphoblastique de phénotype T révélée par une pleurésie purulente et confirmée par biopsie bronchique. Une chimiothérapie a été démarrée avec bonne évolution.

## Introduction

Les Leucémies aigues lymphoblastiques se caractérisent par une prolifération lymphoïde d'installation aigue associée à des cytopénies. Au cours des Leucémies aigues lymphoblastiques les atteintes pulmonaires sont relativement rares, mais l'atteinte ganglionnaire, notamment médiastinale est très fréquente. Les manifestations pulmonaires sont liées à une infiltration lymphatique, avec des lésions nodulaires intra-parenchymateuses fréquemment associées à une atteinte pleurale [1]. Certains cas d'atteinte endo-bronchique ont été signalés, mais ils restent rares. Nous rapportons le cas d'une atteinte endobronchique d'une leucémie aigue lymphoblastique de phénotype T révélée par une pleurésie purulente et confirmée par biopsie bronchique.

## Patient et observation

**Figure 1:**
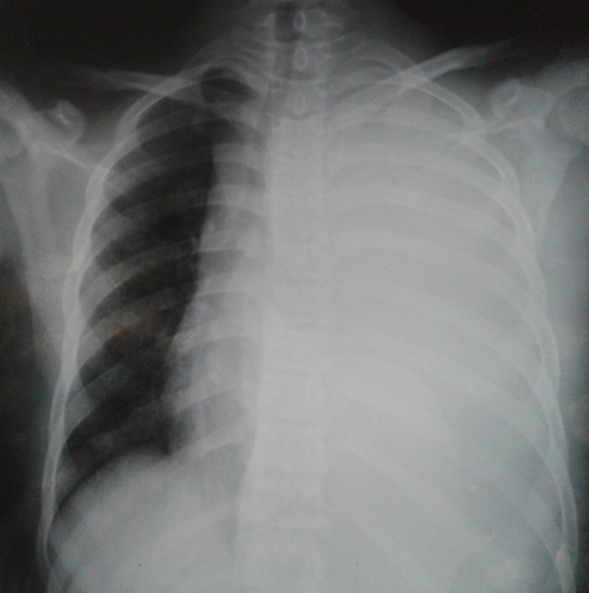
Radiographie thoracique montrant un hémithorax gauche opaque

**Figure 2:**
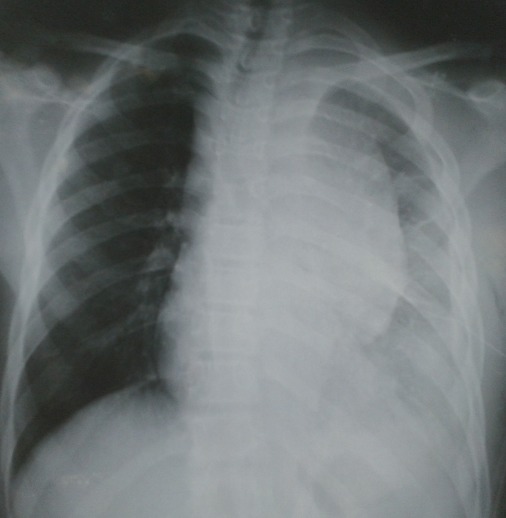
Radiographie thoracique montrant une énorme masse médiastino pulmonaire gauche

**Figure 3:**
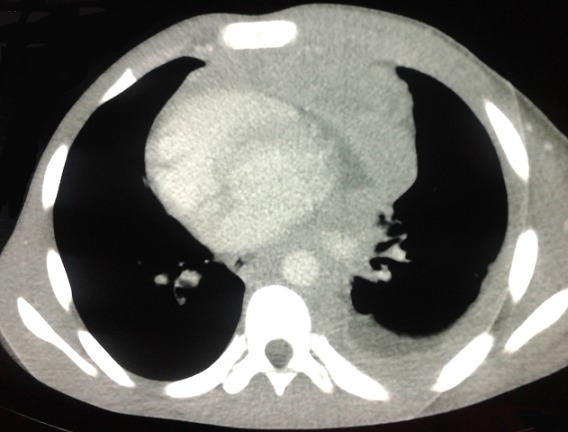
TDM thoracique montrant de multiples adénopathies médiastinales avec un épanchement pleural et péricardique

Mr A.A âgé de 17 ans, agriculteur, sans antécédents particuliers qui a présenté un jour avant son hospitalisation une douleur basithoracique gauche d'installation brutale avec une toux sèche. L'examen clinique a objectivé une fièvre à 39^°^, un syndrome d’épanchement liquidien de l'hémithorax gauche et une adénopathie sus claviculaire gauche de 2cm/2cm. La radiographie du thorax face (Figure 1) a objectivé un hémithorax gauche opaque en faveur d'une pleurésie de grande abondance. Une ponction pleurale a ramené un liquide purulent. Un drainage thoracique avec aspiration continue a été réalisé avec une triple antibiothérapie probabiliste et des séances de kinésithérapie respiratoire. La radiographie thoracique de contrôle après drainage (Figure 2) a révélé une énorme masse médiastino-pulmonaire de l'hémithorax gauche. Un bilan biologique a objectivé une thrombopénie à 56000 /mm^3^, un taux de LDH à 394 UI / L. La TDM thoraco-abdominale (Figure 3) a montré de multiples adénopathies sus et sous diaphragmatiques, un épanchement pleural et péricardique et une hépatosplénomégalie.

**Figure 4:**
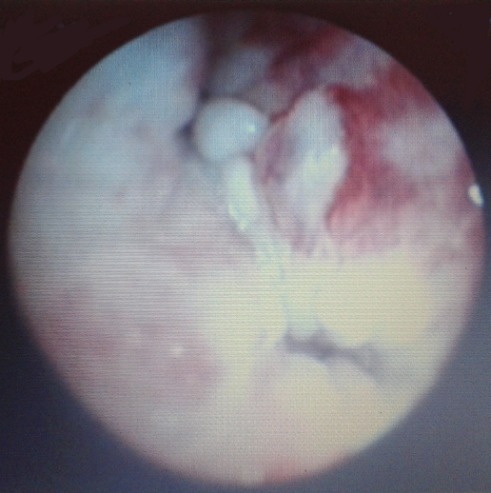
Aspect bronchoscopique montrant de multiples bourgeons d'allure tumorale sténosant l'orifice de la lobaire supérieure gauche

La bronchoscopie (Figure 4) a objectivé une sténose d'allure tumorale avec présence de multiples petits bourgeons au niveau de la lobaire supérieure gauche et à moindre degré de la lobaire inferieure dont l’étude anatomopathologique a révélé une prolifération tumorale maligne à petite cellules rondes. Le complément immunohistochimique a montré un aspect en faveur d'une prolifération lymphoïde maligne diffuse à petites cellules de phénotype T (expression membranaire des anticorps anti-CD10, anti-CD5et anti-CD3). L’étude histologique de la biopsie de l'adénopathie cervicale montre un aspect de prolifération lymphoïde maligne à petites cellules d'architecture diffuse et nodulaire. L’étude cytologique du myélogramme était en faveur d'une leucémie aigue lymphoblastique de phénotype T. Le patient a été transféré au service d'hématologie et traité selon un protocol de MARALL 2006: une phase de corticosensibilité a été démarrée: prédnisolone 60mg/J pendant 8 jours puis une phase d'induction à partir J8 à base de Vincristine, Daunuraubucine, l-asparaginase, immunothérapie triple (Aracytine+ Methotrexate +Corticoïdes) avec bonne évolution.

## Discussion

La leucémie aiguë lymphoblastique (LAL) représente 20% des leucémies de l'adulte. Les manifestations extra-ganglionnaires sont principalement la peau, la rate, l'os, les poumons, la plèvre et le tractus gastro-intestinal [2]. La présentation endobronchique est une forme clinique rare qui survient chez les patients atteints de formes disséminée, quelques cas seulement sont rapportés dans la littérature [3–5]. L'obstruction bronchique cliniquement significative chez les patients atteints de leucémies est généralement le résultat d'une compression par des ganglions lymphatiques péribronchiques, mais rarement peut être la conséquence d'une occlusion par des lésions lymphomateuses endobronchiques ce qui était le cas chez notre patient [1, 6]. Les premières formes des hémopathies lymphoïdes endobronchique ont été discutées dans la littérature par Moolten en 1934, il a décrit des plaques diffuses avec des ulcérations bronchiques, qu'il avait appelé «panbronchitis granulomateuse», dans huit cas d'hémopathie lymphoïdes étudiés en post mortem. Les nodules bronchiques étaient évidents sur l'inspection des grosses voies aériennes dans trois cas, dont l'un était remarquable en raison de l'obstruction complète d'une grande bronche [7].

Divers mécanismes ont été suggérés comme étant responsable du développement de la lésion endobronchique chez les patients atteints des LAL: 1) une invasion directe d'un côté nœud lymphatique médiastinal, 2) l'invasion directe d'une lésion parenchymateuse, 3) la diffusion dans les tissus lymphatiques péribronchiques, et 4) la diffusion hématogène. L’épaississement de la paroi bronchique et l'infiltration sous-muqueuse suggèrent que la diffusion des tissus lymphatiques été impliqué dans la formation des lésions endobronchiques [8]. Le diagnostic de l'atteinte endobronchique est effectué par des examens d'imagerie qui peuvent montrer un collapsus pulmonaire ou la masse endobronchique elle-même, une atteinte ganglionnaire médiastinale associée. La bronchoscopie permet de localiser l'atteinte endobronchique, ce qui était le cas de notre patient, et faire des biopsies bien dirigées pour étude anatomopathologiques, elle permet également de faciliter le traitement local de la maladie permettant d'améliorer la capacité respiratoire des patients [9]. L'infiltration par des cellules leucémiques peut se produire dans de nombreux organes. Cependant, la formation d'une masse tumorale est rare. Green et al, ont rapporté une large série de patients atteints de leucémie de tous types, seulement 4 des 109 avaient une atteinte de la muqueuse bronchique [10]. Les thérapeutiques actuelles reposent sur des stratégies adaptées aux facteurs de risque de la leucémie. L'utilisation de traitements d'induction et de consolidation plus intensifs, éventuellement sous couvert de facteurs de croissance hématopoïétiques, a amélioré les taux de survie. Une prophylaxie d'un envahissement du système nerveux central est également bénéfique, particulièrement en cas de leucémie aiguë lymphoblastique à haut risque. Les leucémies aiguës lymphoblastiques à risque standard sont traitées uniquement par chimiothérapie. Les malades bénéficient, après les traitements d'induction et de consolidation, d'un traitement d'entretien [11].

## Conclusion

L'atteinte endobronchique au cours des leucémies aigues est très rare mais de pronostic péjoratif. Nous insistons sur l'apport de la bronchoscopie dans l'approche diagnostique de cette atteinte.
